# Establishing oral health promoting behaviours in children – parents’ views on barriers, facilitators and professional support: a qualitative study

**DOI:** 10.1186/s12903-015-0145-0

**Published:** 2015-12-10

**Authors:** Denise Duijster, Maddelon de Jong-Lenters, Erik Verrips, Cor van Loveren

**Affiliations:** Department of Preventive Dentistry, Academic Centre for Dentistry Amsterdam, University of Amsterdam and VU University, Gustav Mahlerlaan 3004, 1081 LA Amsterdam, The Netherlands; Department of Cariology, Endodontology and Pedodontology, Academic Centre for Dentistry Amsterdam, University of Amsterdam and VU University, Gustav Mahlerlaan 3004, 1081 LA Amsterdam, The Netherlands; TNO, Schipholweg 77-89, 2316ZL Leiden, The Netherlands

**Keywords:** Qualitative research, Children, Dental caries, Oral health behaviours, Determinants, Interventions, Family, Parenting, Self-efficacy, Routines

## Abstract

**Background:**

The prevention of childhood dental caries relies on adherence to key behaviours, including twice daily tooth brushing with fluoride toothpaste and reducing the consumption of sugary foods and drinks. The aim of this qualitative study was to explore parents’ perceptions of barriers and facilitators that influence these oral health behaviours in children. A further objective was to explore parents’ views on limitations and opportunities for professional support to promote children’s oral health.

**Methods:**

Six focus group interviews were conducted, including a total of 39 parents of 7-year old children, who were recruited from paediatric dental centres in The Netherlands. Interviews were held with Dutch parents of low and high socioeconomic status and parents from Turkish and Moroccan origin. Focus group interviews were conducted on the basis of a pre-tested semi-structured interview guide and topic list. Content analysis was employed to analyse the data.

**Results:**

Analysis of interview transcripts identified many influences on children’s oral health behaviours, operating at child, family and community levels. Perceived influences on children’s tooth brushing behaviour were primarily located within the direct family environment, including parental knowledge, perceived importance and parental confidence in tooth brushing, locus of control, role modelling, parental monitoring and supervision, parenting strategies and tooth brushing routines and habituation. The consumption of sugary foods and drinks was influenced by both the direct family environment and factors external to the family, including the school, the social environment, commercials and television, supermarkets and affordability of foods. Parents raised several suggestions for professional oral health support, which included the provision of clear and consistent oral health information using a positive approach, dietary regulations at school and a multidisciplinary approach among dental professionals, child health centres and other institutions in providing parental support.

**Conclusion:**

In conclusion, this qualitative study provided detail regarding parental views on the influences on children oral health behaviours and their opinions on what further support is needed to promote children’s dental health. Parents’ suggestions for professional oral health support can guide the development or improvement of caries preventive interventions.

## Background

Dental caries is a common childhood disease with a range of biological and behavioural risk factors involved in its aetiology [1]. Children are most likely to develop caries if they acquire *Streptococcus Mutans* at a young age, which can largely be compensated by other parameters, such as good oral hygiene and a non-cariogenic diet [2, 3]. Therefore, the prevention of childhood dental caries mainly relies on adherence to key behavioural messages, including twice daily tooth brushing from an early age with fluoride toothpaste and reducing the frequency of consuming sugary foods and drinks [4]. However, it is increasingly recognized that knowledge of these messages alone does rarely lead to sustained behaviour change in individuals [5]. Simple oral health behaviours are enmeshed in more complex daily habits which are largely determined by a broad scope of psychosocial, economic and environmental factors [6]. In this context, efforts to prevent childhood dental caries cannot narrowly focus on individuals and their biology and behaviours alone, but should consider the underlying determinants of children’s dental health as well.

This increased appreciation has led to articles conceptualizing and exploring the broader influences on the development of childhood dental caries. A comprehensive conceptual model by Fisher-Owens et al. [7] acknowledges a wide range of determinants of children’s oral health and oral health-related behaviours, such as parents’ health beliefs, practices and coping skills [8–10], family functioning [11] and composition [12, 13], social support [11, 14] and more distal factors, such as the living environment [15, 16], culture [17], social capital [18] and the (dental) health care system [19]. These determinants are suggested to operate at both child, family and community level, with interactions occurring across the various levels of influence.

Understanding the determinants of childhood dental caries can be augmented by qualitative research of exploring parental perspectives on the influences of childhood dental caries [20]. Given the role of parents as principal regulators of children’s dietary intake and the important role of the family in shaping children’s oral hygiene habits, it is important to document their views. Also, parents could share their ideas on the guidance that is needed to improve these oral health behaviours. Both are important to consider when developing caries preventive interventions.

There are a few qualitative studies which sought to explore parental perspectives on children’s dental health promoting behaviours [17, 21–28], yet these studies mainly focussed on influences on children’s tooth brushing behaviours alone or attitudes towards the significance of children’s dental health. Furthermore, no studies have been conducted to investigate parents’ views on current caries preventive interventions and opportunities for improvement. Therefore, the present study conducted focus group interviews with parents of 7-year-old children from The Netherlands, with the aim to explore their perceptions of factors (barriers and facilitators) that influence children’s oral health behaviours. Focus groups were chosen as a method, as opposed to individual interviews, because it encourages parents to provide open responses and it allows parents to build on each other’s ideas through facilitated discussion. The oral health behaviours studied were twice daily tooth brushing with fluoride toothpaste and reducing children’s consumption of sugary foods and drinks. A further objective was to explore parents’ views on limitations and opportunities for professional support to promote children’s oral health.

## Methods

### Ethical approval

Approval for this study was obtained from The Medical Ethical Committee of the VU University of Amsterdam, The Netherlands (registration number 2012/144). Participating parents were informed via a postal letter that they were free to withdraw from the study at any time and that all data would be handled with full regard to confidentiality and anonymity. Parents were notified that they were recruited on the basis of sociodemographic factors and their child’s oral health status. These data had been collected in a study in which they had previously taken part [11]. Prior to data collection, all participating parents provided written informed consent to participate in the study and to use the data for publication.

### Study population background

In the Netherlands, healthcare is based on a single compulsory health insurance scheme. Dental health care is provided by private dentists and dental hygienists. All dental care for children under 18 years of age is automatically covered under parents’ health insurance premium. Most children are registered with a dentist, but this is not mandatory.

Key messages in Dutch oral health promoting guidelines are twice daily tooth brushing with fluoride toothpaste and restricting the consumption of foods and drinks to a maximum of 5 to 7 times a day.

### Study design and sampling procedure

Qualitative focus group interviews were conducted between November 2012 and July 2013. Participants were parents of children who had previously taken part in a quantitative cross-sectional study in 2011–2012, which was set up to investigate family-related determinants of childhood dental caries [11]. In this quantitative study, a stratified random sample of 630 6-year old children was recruited from paediatric dental centres located in various regions in The Netherlands. Data on sociodemographic characteristics were collected using parental questionnaires and children’s dental health status, expressed as the number of decayed, missing and filled teeth (dmft score), was extracted from personal dental records.

For the present study, a purposive sampling technique was used to select a subgroup of parents to participate in the focus group interviews. Selection was based on parents’ ethnic background, socioeconomic status (SES), geographical region and their child’s dental health status to ensure that a diverse range of views was adequately represented. Homogeneous focus groups of people from similar cultural and socioeconomic characteristics were created, because homogeneous groups are generally more comfortable and open with each other, whereas mixed ethnic or socioeconomic groups make it more difficult to achieve a high degree of group interaction [29]. Separate focus group interviews were held with parents who were born in The Netherlands, parents who were first-generation immigrants from Turkey, and parents who were first-generation immigrants from Morocco. These two latter ethnic groups were targeted, because they constitute 12-20 % of the population in the larger cities in The Netherlands [30], and the caries prevalence among children from these ethnic groups is relatively high [31]. Focus groups with Dutch parents were stratified by SES. The mother’s highest completed level of education was used as an indicator for SES, which categorized parents into a low SES group (no education, elementary school, secondary school at lower level and further education at lower level) and a high SES group (secondary school at higher level, further education at higher level and University). The focus groups with Turkish and Moroccan parents were not stratified by SES, because the vast majority of first-generation immigrants from Turkey and Morocco that participated in the quantitative study were from low SES as determined by their education level. Furthermore, within each focus group, parents of caries free children (dmft = 0), parents of children with moderate levels of dental caries (dmft ≥ 1 < 4) and parents of children with high levels of dental caries (dmft > 4) were purposively selected. Focus group interviews were held in four different geographical areas in which a paediatric dental centre was located, namely in Zoetermeer, Enschede, Den Haag and Utrecht. The areas vary greatly in terms of socioeconomic location and the proportion of immigrants living in the area.

All selected parents were informed about the study by telephone and requested to participate. Parents who agreed to participate received a confirmation letter at their home address, informing them about the aim, procedure and appointment details of the study. Only one parent per family was requested to take part. A monetary voucher of 25 euro’s was given as an incentive to participants.

### Data collection

A semi-structured interview guide was developed to ensure consistency in data collection among focus group interviews, yet allowing the sessions to be flexible to optimize the natural flow of conversation in the groups. The interview guide included a series of open-ended questions to reduce the chance of priming and bias. The questions were designed to elicit discussion among parents about factors they perceived to influence children’s oral health behaviours (i.e. twice daily tooth brushing and reducing the consumption of sugary foods and drinks), and to stimulate discussion about what further (professional) support they think is needed to promote children’s oral health. Examples of questions were: ‘Could you describe your experiences with brushing your child’s teeth?’, and ‘What were things that made it either easy or difficult to brush your child’s teeth?’. A topic list, based on scientific literature and Fisher Owens’ theoretical model of children’s oral health determinants [7], was used to guide the interviews. Topics included potential influences on children’s oral health behaviours, such as child temperament, child preferences, routines, time, family composition and division of family roles, parenting, parental stress, parental depression, social support, peer pressure, health care, media and advertisement, schools and the availability and affordability of resources. Topics of the list were only introduced in the focus group interviews when they were not spontaneously brought up in the discussion. The questions were pilot-tested for clarity, comprehension and suitability in one focus group interview with parents working at ACTA, department of ‘Social Dentistry’ and one with Turkish and Moroccan students. The interview guide is available upon request.

The focus group interviews were performed in a quiet room at a paediatric dental centre, and lasted between 75 and 120 min (mean time: 100 min), including a 15-minute break. All focus group interviews were conducted by a moderator (DD, MSc in Dental Public Health, PhD-student and trained in conducting qualitative research), who guided the discussion, and an assistant moderator/observer (MdJL, MSc in Paediatric Dentistry, PhD-student and working as a paediatric dentist), who took field notes and made sure that all participants contributed to the discussion. All focus group interviews were audio-recorded and transcribed verbatim. The audio tapes, transcripts and other supporting data were stored digitally in a password protected database at the Academic Centre for Dentistry Amsterdam, which was only accessible for the authors (DD and MdJL).

### Data analysis

Thematic analysis was employed to analyse and interpret the content of the data [32]. First, open coding was done through reading the transcripts and assigning codes line by line, forming the initial coding scheme. Secondly, related codes were sorted and clustered to identify themes. Fisher-Owens’ theoretical model of children’s oral health determinants [7] was used to guide the thematic data analysis and to structure the identified themes into child level influences, family level influences and community level influences according to Fisher-Owens’ model of. MAXQDA (software for qualitative data analysis, 1989–2014, VERBI Software - Consult - Sozialforschung GmbH, Berlin, Germany) was used to manage the data analysis.

The open coding of all transcripts was performed by one author (DD). All authors fully read the transcripts. The initial coding scheme and the identified themes were evaluated and discussed in various group sessions with the remaining authors (MdJL, EV and CvL) until consensus was reached, For reporting purposes, quotes were translated from Dutch to English by a bilingual person (DD). The consolidated criteria for reporting qualitative research (COREQ) checklist was used to ensure quality in the reporting of this study [33].

## Results

### Characteristics of focus groups and participants

Six focus group interviews were conducted, including two focus group interviews with Dutch parents of high SES, two focus group interviews with Dutch parents of low SES, one focus group interview with Turkish parents and one focus group interview with Moroccan parents. A total number of 39 parents participated in the study (response rate 36 %), ranging from 4 to 10 parents per focus group session. The response rate in the Turkish and Moroccan group was somewhat lower, due to language barrier and difficulties with transportation to the dental care centre. The mean age of the child of selected parents was 7.2 ± 0.5 years. The characteristics of participants are described in Table 1.

**Table 1:** Characteristics of participants per focus group interview

Variables		D-HSES-1	D-LSES-1	D-HSES-2	D-LSES-2	T^a^	M	Total
		(*n* = 10)	(*n* = 8)	(*n* = 4)	(*n* = 5)	(*n* = 6)	(*n* = 6)	(*n* = 39)
		*n*	*n*	*n*	*n*	*n*	*n*	*n (%)*
Sex of the child								
	Girl	7	2	1	4	2	2	18 (47.4)
	Boy	3	6	3	1	3	4	20 (52.6)
Dental health status of the child								
	Dmft = 0	5	4	3	3	2	1	18 (47.4)
	Dmft ≥ 1 < 4	4	3	-	1	2	3	13 (34.2)
	Dmft > 4	1	1	1	1	1	2	7 (18.4)
Participating parent								
	Mother	9	8	4	5	2	3	31 (79.5)
	Father	1	-	-	-	4	3	8 (20.5)
Education level of the mother								
	University	3	-	-	-	-	-	3 (7.9)
	Further education (higher level)	4	-	4	-	-	-	8 (21.1)
	Secondary school (higher level)	3	-	-	-	-	-	3 (7.9)
	Further education (lower level)	-	7	-	3	-	1	11 (28.9)
	Secondary school (lower level)	-	1	-	2	3	1	7 (18.4)
	Elementary school	-	-	-	-	2	2	4 (10.5)
	No education	-	-	-	-	-	2	2 (5.3)
Relationship status of the parent								
	With partner	10	6	4	4	3	5	32 (84.2)
	Single	-	2	-	1	2	1	6 (15.8)
Number of siblings per household								
	0 – 1 sibling(s)	8	6	1	3	2	2	22 (57.9)
	≥2 siblings	2	2	3	2	4	3	16 (42.1)

*D-HSES-1 and D-HSES-2* focus group interviews with Dutch parents of high SES, D-LSES-1 and *D-LSES-2* focus group interviews with Dutch parents of low SES, *T* focus group interview with Turkish parents, *M* focus group interview with Moroccan parents

^a^For one child, both the father and mother participated in the focus group session

### Structure of the results section

The results section contains two sections. First, the results on parents’ views on children’s oral health behaviours are described, which are broken down into two parts: ^a.^ ‘twice daily tooth brushing with fluoride toothpaste’, and ^b.^ ‘controlling the consumption of sugary foods and drinks’. The second section reports on parents’ views on limitations and opportunities for professional oral health support. The themes for each section are described in the context in which they were discussed in the focus group sessions, and they are illustrated with interview quotes of parents (sentences in italic).

- Parental views on influences on children’s oral health behaviours
- Twice daily tooth brushing with fluoride toothpaste

**Fig. 1:**
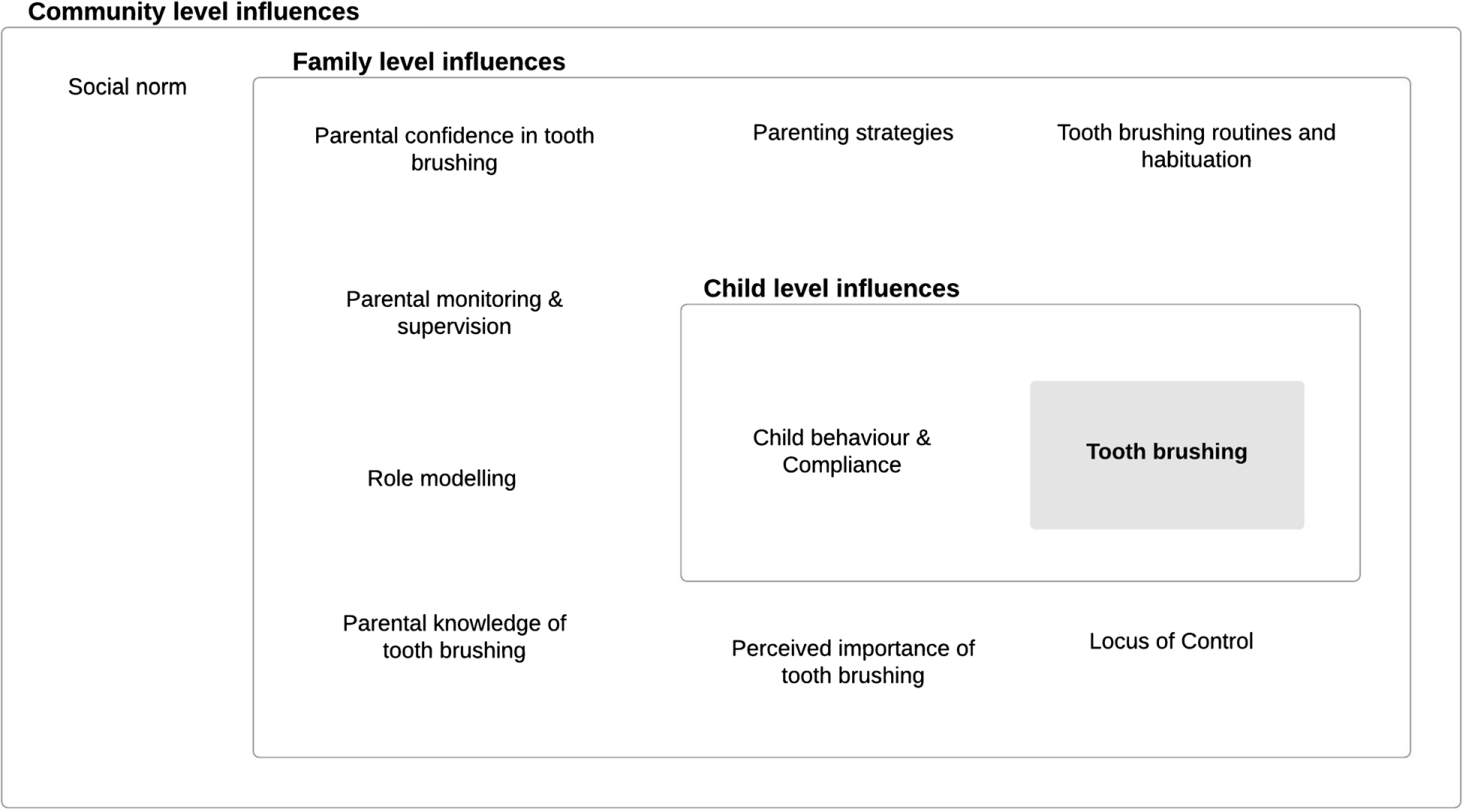
Parental views on factors influencing twice daily tooth brushing with fluoride toothpaste in children

Analysis of the focus group interviews identified ten themes of influences on children’s tooth brushing behaviour. These are schematically presented in Fig. 1, in which influences are mapped to child, family and community levels.

#### ‘Social norm’

Parents perceived twice daily tooth brushing as a generally accepted standard of behaviour (social norm). Social norms refer to the extent to which individuals think that others in their network or community practice a certain behaviour (e.g. tooth brushing). In addition, it requires individuals to believe that people in their network or community think it is important that they also practice the behaviour themselves.

#### “Perceived importance of tooth brushing” and “locus of control”

In general, parents acknowledged the value and importance of twice daily tooth brushing with fluoride toothpaste to maintain good oral health for their child. In most families parents managed to brush their children’s teeth twice a day, usually in the morning before or after breakfast and in the evening before bedtime. However, many of the parents whose children had caries experience did not believe that oral hygiene efforts could fully prevent their child from getting tooth decay, defined as an external locus of control. They often related childhood dental caries to causes outside the parent’s and child’s control, such as chance, genetics or health problems in childhood. A low-SES Dutch mother said: *“It must be the genes of my husband, because my teeth are fine…”* and a Moroccan father said: *“When my son was 4 years old they had to extract six teeth. I think his teeth were bad because he’d been given lots of antibiotics for asthma when he was young”.*

#### ‘Child behaviour & compliance’, ‘parental confidence in tooth brushing’ and ‘parenting strategies’

Many parents expressed that they felt confident in their ability to successfully establish twice daily tooth brushing for their child, indicating they had a high dental self-efficacy. A high-SES Dutch mother said: “…*It’s just perseverance. Her teeth are always brushed twice a day”.* In the course of the interviews, however, many parents described situations in which they experienced difficulties with tooth brushing. A common barrier was associated with difficult child behaviour and non-compliance in response to tooth brushing. Some parents stated that it was sometimes a struggle to brush their child’s teeth, e.g. due to resistant behaviour, tantrums, pain during teething or tiredness of the child. A low-SES mother said: *“For a time period I had this strong-willed toddler who was convinced he could do it all by himself. He just wouldn’t allow me to brush his teeth for him”.* A few parents described that they sometimes rather avoided conflict in those situations, than to persist on tooth brushing A Moroccan mother said: *“When he’s uncooperative in the morning I’m not always going to battle with him. Certainly not me, no.”* Other parents reported various parenting strategies to cope with children’s non-compliant behaviour towards tooth brushing. Some parents tried to maximize compliance using positive reinforcement (e.g. giving compliments or providing rewards, such as a sticker or new tooth brush) or by turning tooth brushing into an easy/enjoyable activity (e.g. singing a song, using a tooth brushing poster with icons, setting an alarm, counting along). A high-SES Dutch mother said: *“For a while it was a real struggle to brush her teeth, until we let go a little and tried to make it more positive by giving compliments”.* Another high-SES Dutch parent reported that she used disciplinary restrictions, such as withholding privileges, to realize twice daily tooth brushing. A few parents used rigid disciplinary strategies by physically restraining the child to ensure that tooth brushing was properly performed. A low-SES Dutch mother said: *“…I just held her in head lock for two minutes…”.* Moreover, many parents agreed that it is essential to be consistent when disciplining their child. A high-SES Dutch mother said: “*Eventually, you are the boss. I believe it’s very important not to give in to your child, because then it will always try to push boundaries”.*

#### ‘Tooth brushing routines and habituation’

In each focus group interview, many parents agreed that routines and structure in the family were very important to manage twice daily tooth brushing in children. A high-SES Dutch mother said: *“Some children are always ten minutes late at school because their families don’t have routines and structure. These are often the same children that haven’t had breakfast and haven’t brushed their teeth”.* Many parents reported that tooth brushing was embedded into a ritual of routinized daily activities, such as washing and getting dressed. Habituation helped to successfully implement the behaviour: A Turkish mother said: *“I’ve never perceived tooth brushing to be difficult because it’s such an automatism. The children are just used to it”.*

In each focus group interview, a few parents admitted that they sometimes skipped brushing their child’s teeth due to time constraints or a busy schedule. Tooth brushing in the morning was considered more challenging than in the evening. A low-SES Dutch mother said: *“Mornings are often busy, especially when we both have to go to work. It needs planning. We’re in a hurry to brush their teeth and then the brushing is not always done very thoroughly”* and another mother from the low-SES group said: *“I don’t have time to brush their teeth in the morning. I mean… I leave at 7 am and I have to dress two children, make breakfast for them, and so on. Of course I have a partner, but he’s like; Ah, don’t worry…”.* To facilitate tooth brushing in the morning, a few parents had placed an extra toothbrush downstairs, so that after breakfast children did not have to go upstairs to brush their teeth.

#### ‘Role modelling’ and ‘parental monitoring and supervision’

Many parents said they intended to monitor their child’s tooth brushing routines, either by brushing their child’s teeth for them, by re-brushing their child’s teeth or by supervising the child during brushing. A low-SES Dutch mother said: *“First, he gets to brush by himself and then I re-brush his teeth. That’s something I really try to pursue.”* A few parents perceived that brushing their own teeth in their child’s presence encouraged the child to brush too, by functioning as an example or role model for their child. A few parents mentioned not to supervise their children’s tooth brushing habits: A high-SES Dutch mother said: *“I’m not around when they brush their teeth. I am already downstairs when they’re in the bathroom, so I have no clue how well they are brushing their teeth”.*

Many parents reported greater involvement in their children’s oral hygiene when children were young, which helped to control the behaviour. With growing age, children were considered more autonomous and more responsible for their own dental health, resulting in less parental involvement and monitoring. Another high-SES Dutch mother said: “*When they are young you help them with everything, including tooth brushing. As they get older and more independent, they can brush their own teeth, and then you have to be very careful that those two minutes don’t become 1, 2, 3…10, done!”.*

#### ‘Parental knowledge of tooth brushing’

Some parents were insecure about details of knowledge concerning tooth brushing, which became apparent from questions they raised during the interviews (e.g. the best type of tooth brush, the recommended age to allow children to brush by themselves, etc.). Some parents had been given complicated advice, such as ‘not to brush within 30 min after eating or drinking’, or ‘not to brush before breakfast’ or ‘to be careful about the child swallowing toothpaste’, which made it difficult to adhere to advice.

b. Controlling the consumption of sugary foods and drinks

**Fig. 2:**
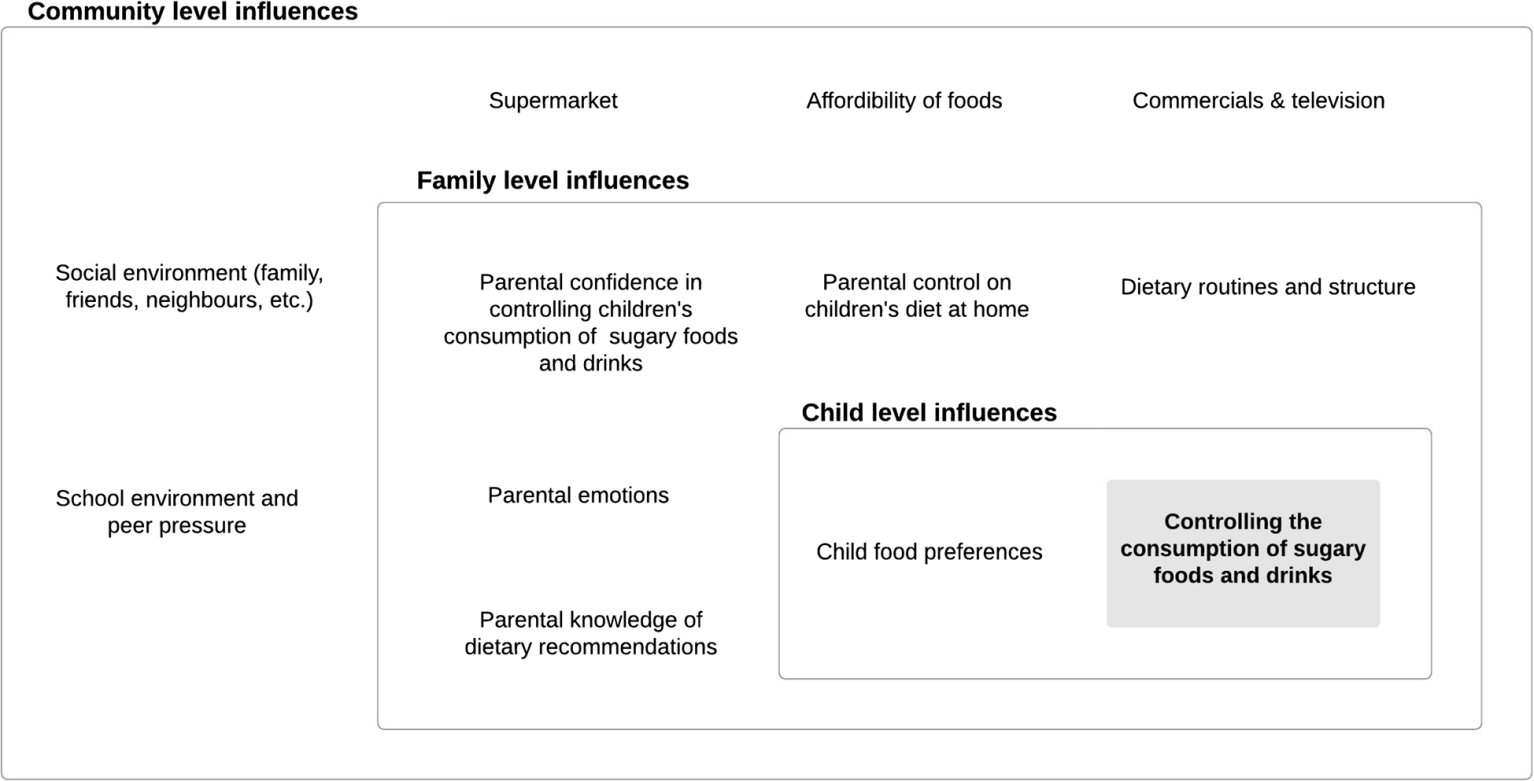
Parental views on factors influencing children’s consumption of sugary foods and drinks

Analysis of the focus group interviews identified eleven themes of influences on children’s consumption of sugary foods and drinks. These are schematically presented in Fig. 2 and they are mapped to child, family and community levels.

#### ‘Parental knowledge of dietary recommendations’

Many parents recognized the importance of reducing children’s intake of sugary foods and drinks to prevent dental caries in children. Most of these parents had been given advice to limit the frequency of sugar consumption to a maximum of 5 to 7 times a day. However, there was quite some confusion among parents which foods and drinks are considered ‘unhealthy’ for their child’s teeth. A high-SES Dutch mother said: *“Yes, but what are sugary foods? I mean… Is a multigrain biscuit also considered ‘sugary’?*”. Furthermore, a few parents had been given dietary advice by their dentist that was in conflict with dietary messages that are important for their child’s general health and development: Another mother of the Dutch high-SES group said: *“One of the things the dentist told me is that fruits can be bad for your child’s teeth. So you think you are doing it right by giving your child healthy foods, and then it turns out…”.* These unclear and conflicting messages were perceived as barriers to adhere to the advice.

Many parents intended to control their child’s intake of sugary foods and drinks, not only to maintain good oral health for their child, but also from a general health perspective. However, there was also a number of parents, especially in the Turkish and Moroccan focus group, who were not concerned about their child’s diet from an oral health perspective, because they did not believe that sugary foods and drinks were damaging for their child’s teeth. A Turkish father said: *“It’s often attributed to sugars and sweets, but that’s just nonsense!”.*

#### ‘Child food preferences’

Parents cited that the difficulty of controlling children’s sugar consumption partially depended on child-related factors. Preference for certain foods and drinks (e.g. having a sweet tooth) and children disliking healthy foods or being ‘picky eaters’ were considered barriers to realising a healthy diet.

#### ‘Parental confidence in controlling the consumption of sugary foods and drinks’ and ‘Parental emotions’

Some of the parents reported that they felt efficacious in controlling their child’s intake of sugary foods and drinks. They were confident about their ability to provide a healthy diet by giving their child healthy foods and drinks to school, by restricting the daily frequency of consuming sugars and by providing healthy alternatives when their child asked for sweets. A low-SES Dutch mother said: *“If they are really hungry I tell them to eat an apple or a cracker with cheese. At least that’s a little justified”*. However, many parents admitted that they did not always feel competent to adhere to dietary advice given by their dentist. Some of the parents believed that the advice was infeasible. A common barrier was related to coping with children’s behaviours, wishes and conflict, for example, when children kept asking for sweets. A Turkish mother said: *“…Of course it sometimes happens that I give in to my child when she keeps nagging for sweets. Obviously. We’re humans, right?”*. Parental emotions also played a role. Some parents felt guilty to give their child healthy foods and drinks that they dislike. A mother from the low-SES group said: *“I would feel very sorry for him when he opens his mug at school and he would see it has water in it…”.*

#### ‘Dietary routines and structure’ and ‘parental control on a children’s diet at home’

Parents differed in opinion about the difficulty of reducing the frequency of sugar consumption at home. Mainly the parents with a high confidence (especially in the focus groups with Dutch parents of high SES) experienced little difficulty in controlling children’s intake of sugary foods and drinks when they are indoors. Perceived facilitators included family structure and parents’ ability to monitor their child’s dietary intake at home. Many of these parents said to have a regular and routinized daily eating pattern. A low-SES Dutch mother said: *“It’s a standard routine. They have breakfast in the morning, they have one healthy snack and lunch at school, and after school they have one more snack or piece of fruit before dinner. That’s it…”.* Having family meals together was believed to add structure to children’s dietary patterns. The same parents also reported to have clear rules and agreements at home about sugar-snacking. A Dutch mother from the high-SES group said: *“It’s very easy. They know when they can have a snack or sweets. They are familiar with the rules and, I mean, there is just no debate about that”* and another mother added: *“They are not allowed to take snacks from the kitchen cupboard”.* Many of these parents also agreed that parental monitoring helped to control their child’s sugar consumption at home, because they were able to supervise children in their direct presence.

In contrast, there were also many parents (more often in the focus groups with parents from lower SES and ethnic groups) who perceived barriers to limit their child’s consumption of sugary foods and drinks at home, in particularly in the weekends. These parents described less daily structure and less clarity and consistency of rules and agreements about sugar-snacking. A low-SES Dutch mother said: “*In the weekend …Oh well, than I also take something when I have an appetite for food”* and a Turkish mother said: *“He just gets candy or cookies when he asks for it and I think; Yes, now it’s ok”.* Furthermore, many of them reported situations at home in which they felt unable to monitor their child’s diet, e.g. when they are not in their child’s direct presence or when they can’t pay attention because they are occupied with other activities at home. A high-SES Dutch mother said: *“In the weekends, it often happens that they wake up earlier than we do, and then they’ve already had a cookie-breakfast”.*

#### ‘Social environment (family, friends, neighbours, etc.)’

Many parents agreed that they had little control over their child’s dietary intake when children were outdoors. Some of the parents reported that children were often indulged with sweets and snacks when visiting neighbours, grandparents or food shops, such as the bakery or butcher. A high-SES Dutch mother said: *“Grandparents are the worst of course. It’s unbelievable how much food they get when they’re visiting. Always lots of crisps and sweets during the day… And often my mother also puts down a bowl of candy in the evening. She just likes to spoil them”.* In case of an exception, this was often not seen as a problem. However, parents expressed concern when children frequently visited friends or family where they applied different norms and rules about sugar-snacking, or if someone who regularly looked after the children used different rules. A low-SES mother said: “*My mother was often babysitting and I found it very hard to ask her to follow my rules and advice, because I didn’t want to offend her…”.*

#### ‘School environment and peer pressure’

Many of the parents who felt it was relatively easy to control children’s sugar intake at home reported many barriers to ensure a healthy diet at school. They perceived a lot of peer pressure from other parents who gave their child sweets or unhealthy snacks to school. A high-SES Dutch mother said: “*I’ve seen what parents give their children to school; chocolate bars, almond cakes, it’s shocking!”* and a Moroccan mother said: *“It’s not mandatory to give your child a snack to school, but the 10 ‘o clock snack-break is obviously a very social thing. And of course it’s not very nice for him when he’s the only one who doesn’t have something yummy”*. Yet, other parents, mainly those who reported barriers at home, expressed the belief that school helped to limit children’s sugar consumption due to routinized structure and dietary regulations at school. A Moroccan father said: *“On weekdays it’s much easier to reduce the number of eating and drinking moments, because at school they have fixed mealtimes. It’s just routine”* and a Dutch mother from the low-SES group said: *“At our school we have a newsletter in which parents are explicitly advised to give children fruit or a vegetable snack to school”*.

Birthday treats were seen as a barrier to ensure a healthy diet at school. Furthermore, a few parents expressed concern about children’s increasing autonomy with growing age. A high-SES Dutch mother said: *“I don’t want to know what’s going to happen when they’re going to high school. I mean… the gulls know exactly at what time children have lunch break… Children throw their sandwiches into the trash bin and they use their pocket money to buy their own food at the school canteen…”*.

#### ‘Supermarket’, ‘commercials & television’ and ‘affordability of foods & drinks’

A few parents acknowledged the impact of commercials, television and supermarkets on children’s dietary wishes, however, many of them said this did not influence their purchasing behaviour, or only on exceptional occasions. A Turkish mother said: *“…They’re certainly influenced by commercials. That’s where they get their ideas from, as well as from their classmates. They often come with suggestions ‘Mom, I’ve seen this, can you buy that next time?’. I sometimes do when it’s a holiday for example”.*

A Dutch mother from the low SES-group said that prices influenced what foods and drinks she bought for her children, but not in a health adverse way: “*I live on a very tight budget, so I really have to be cautious with how I spend my money. First I buy the things I need, such as fresh fruits, vegetables, bread and meat, and if I have money left I can buy extra’s, such as potato chips or chocolate eggs for Easter*”.

2. Parental views on limitations and opportunities for professional oral health support

Parents were encouraged to give their opinion about current oral health interventions in general and they were asked what further support they think is needed to promote children’s oral health. Responsibilities and opportunities for oral health support were identified at four professional and institutional settings: dental professionals, child health centres, school and Kindergarten and other institutions, including social welfare and health insurance companies. Table 2 presents an overview of perceived limitations and suggested support per health profession/institution.

**Table 2:** Parental views on limitations of and opportunities for professional support to promote children’s oral health

Setting	Perceived limitations	Opportunities
Dental professionals	Little priority for prevention and advice	Encouraging dental visits at an early age
	Limited involvement of parents	Delivering dental health education in group discussions
	Dissatisfaction with content of dental health education:	Improving the content of dental health education:
	• Insufficient and very general information • Complicated and conflicting messages	• Simple, clear and consistent messages • Tailored advice
	Dissatisfaction with delivery of dental health education:	Improving the delivery of dental health education:
	• Tone	• Increasing attention and expression of understanding
Child health centres	Little priority for oral health promotion	Referring to a (paediatric) dentist at an early age
	Dissatisfaction with content of dental health education:	Integrating dental health education into general consultation visits (e.g. by assistant in waiting room)
	• Insufficient and very general information • Complicated and conflicting messages (oral health and general health)	Providing information leaflets or showing video’s in waiting room
Schools	Age of children: late advice and prevention	Delivering dental health education at schools
	School dental health education: no long term effect on behaviour change	Organizing theme projects at schools
		Implementing dietary regulations at schools
		Promoting fruit days at schools
Kindergarten		Organizing tooth brushing group activities: learning by doing
		Delivering dental health education to parents in group discussions at the day care centre
Social welfare		Collaborating between dental professionals and social welfare: providing parenting support
Health insurance companies	Commercial interests	Providing information leaflets and oral hygiene aids
	Privacy issues	Providing lists of dental practices in the area

#### ‘Dental professionals’ and ‘Child health centres’

The provision of oral health support was mainly considered the responsibility of dental professionals, since it’s their area of expertise. Parents also said that the role of child health centres in caries prevention could not be ignored, as they reach a large proportion of the population, because families regularly visit the centres for child health and growth monitoring from an early age. Suggested tasks for child health centres included the provision of dental advice to novice parents and to ensure timely visits to a dental practice.

Many parents reported that (dental) health care professionals spend little time on informing parents about caries prevention, and they felt that attention and subsidies for public oral health promotion has decreased over the years. Many parents expressed the desire to receive proper oral health information, starting early in a child’s life. Common requests were to receive clear and tailored advice and practical tips to help the implementation of dentally healthy behaviours for their child (e.g. using stickers as a daily incentive or an alarm to facilitate brushing, placing a tooth brush downstairs, receiving tips on non-cariogenic snacks, etc.). They also stressed that they wanted to feel heard and supported by the person providing the health information, rather than feeling blamed. A high-SES Dutch mother said: *“It’s so frustrating when you get an accusing comment, such as ‘Are you really re-brushing his teeth?’ Right away! It would be much more helpful if they say ‘Well, this is already going ok, but this could need some attention’. A positive approach, you know…”.* Some parents preferred to receive health information in group sessions, as they reported positive experiences. Another high-SES Dutch mother said: *“I really enjoyed the health information sessions that they organized at the dental centre. I really learned a lot and I’m still benefitting from them”.*

#### ‘Schools’ and ‘Kindergarten’

Parents also discussed opportunities to improve children’s oral health behaviours via schools. Some parents suggested that oral health education at schools or theme projects about oral health may be useful to raise awareness about oral health in children. However, there were also some parents who questioned the long-term benefit of school health education, especially when parents were not involved. A low-SES Dutch mother said: *“… No, that’s only temporary. You can’t expect children to start brushing their teeth until they’re 6, 7 or 8 years old after only one class. That’s really up to the parent to get that done”.* Other recommendations at schools involved the introduction of fruit days and the implementation of dietary regulations, e.g. prohibiting sugary snacks during break time.

A few parents suggested organizing tooth brushing group activities at Kindergarten. Kindergarten was also seen as an opportune setting to target parents for early dental advice in group sessions.

#### ‘Social welfare’

A few parents discussed the option of collaborating with institutions, such as social welfare or youth care services, to provide parental support for families who experience multiple difficulties with raising their child: A high-SES Dutch mother said: *“If a parent doesn’t succeed to get his or her child’s teeth brushed then there might be more problems concerning parenting in general. Perhaps social welfare could provide help in these situations, because this is beyond the ability of the dentist”.*

#### ‘Health insurance companies’

The potential role of health insurance companies in promoting children’s oral health behaviours was also discussed. Some parents raised concerns due to privacy issues and possible conflicting commercial interests. However, other parents said they appreciated to receive information leaflets with age-focused dental advice, e.g. complemented with oral hygiene aids. Also, a list of (paediatric) dental practices in the area was welcome.

## Discussion

This qualitative study provided an elaborate description of the influences on children’s oral health behaviours from the perspective of parents of 7-year old children from The Netherlands. Two models were introduced which include barriers of and facilitators to the adherence of twice daily tooth brushing with fluoride toothpaste and controlling the consumption of sugary foods and drinks. Furthermore, parents were asked to give their opinion about limitations and opportunities for professional support to promote children’s oral health.

This was one of the first studies that used a comprehensive qualitative approach to explore both parents’ perceptions on the determinants of children’s key oral health behaviours, as well as their views and ideas for professional oral health support. Previous qualitative studies in the dental literature solely focussed on tooth brushing behaviour [21–23] or beliefs, attitudes and practices regarding children’s oral health in general [24], or they referred to specific population groups (e.g. cultural groups and children treated under general anaesthesia) [17, 25–28]. A strength of this study is that the qualitative data allows for in depth exploration of the indirect processes that are involved in the adoption of behaviours, while quantitative data is often restricted to exploring direct associations between predetermined and measurable variables.

However, findings of the current study should be considered in the light of some limitations. First, the generalizability of findings is limited by the qualitative nature of the study. Not all views may have been adequately represented due to a high non-response rate and selection bias, because parents of children who visit a regular dental practice and Turkish and Moroccan parents who do not speak the Dutch language were not included. However, in the six focus group interviews in this study thematic saturation was reached [34], meaning that additional participants would likely not have added new information enriching the depth or scope of the data. Secondly, parental responses may have been influenced by the opinions and perceptions of more vocal parents, although this was obviated to a certain extent by the assistant-moderator. In addition, the choice of location at the dental centre may have increased the risk of parents responding in a socially-desirable manner.

Perceived influences on children’s tooth brushing behaviour were primarily located within the direct family environment The role of parents and the family as mediators/moderators of children’s oral health behaviours is also increasingly acknowledged in the dental literature [35]. In terms of children’s consumption of sugary foods and drinks, also many extra-familial factors were felt to be of influence, including the school, the social environment, commercials and television, supermarkets and affordability of foods. This concurs with findings from the obesity literature on factors influencing children’s dietary behaviours in general [36]. Although generally the same themes of influences were discussed in each focus group interview, parents in the Turkish, Moroccan and low SES focus groups more often perceived genetics to play a role in caries aetiology and they often identified barriers within the direct family environment, while parents from high SES focus groups more commonly reported barriers at school or the social environment. However, the qualitative design of this study does not allow quantification of differences between ethnic and social groups, but the findings indicate that this is an important area that needs more examination in quantitative research.

The focus group interviews also revealed information on the limitations that parents experienced with current oral health interventions and their opinions about further support that is needed to help parents in establishing good oral health for their children. Parents discussed limitations and opportunities at multiple professional disciplines, namely dental professionals, child health centres, schools and Kindergarten, and other health institutions. Their suggestions for improvement concerned the desire to receive clear and tailored oral health information, starting from a child’s early age. Perceived obstacles referred to the complexity of current advice and the wide diversity in recommendations, which were sometimes also in conflict with recommendations received elsewhere. A recent review also highlighted a wide difference in recommended tooth brushing methods by dental associations, professionals, companies and texts [37]. This is a serious concern to all (dental) health professionals. This highlights the urgent need for achieving consensus on clear, simple and evidence-based oral health recommendations, both within the dental profession and between all disciplines that could play a role in children’s oral health, e.g. by broad implementation of guidelines. Another complaint of parents was that oral health advice was often delivered using a victim-blaming tone. Parents indicated to be more susceptible for advice if they felt positive involvement and understanding from health professionals.

However, while dental health information can be a prerequisite to engage in dentally healthy behaviours, there is limited evidence for the effectiveness of a purely educative approach in achieving long term behaviour change [5, 38]. The current study demonstrated that many parents in the focus groups possessed sufficient oral health knowledge and motivation, but they still reported many barriers to adhere to the advice. This suggests that where parents accept preventive health messages, many need support in implementing them. However, the suggestions that parents raised for professional oral health support were minimally related to the barriers they experienced within their own family. Thus, there was a clear discrepancy between the perceived ‘problems’ and the suggested ‘solutions’. Therefore, for future qualitative research, it would be interesting to ask parents directly how they think that each reported barrier could be addressed in interventions.

Many reported barriers in this study seemed to revolve around the family environment. Therefore, a family-based approach may be effective in dental caries prevention, which focusses on active parent involvement in children’s disease prevention, targeting multiple family members rather than that of the child alone [39]. Those interventions may include components to improve parents’ dental self-efficacy and beliefs, and training of parenting skills (e.g. positive reinforcement, child management) and habit formation (e.g. establishing daily routines). As parental support cannot only rest with dental health professionals, a multidisciplinary approach seems necessary. This view was shared by general dental practitioners from the United Kingdom, who felt isolated in their efforts to promote the oral health of high risk children and reported the need to broaden the involvement of partners from primary care settings [40]. Integrated care requires short links and clear communication lines between i.a. dental professionals, child health centres, school teachers and social welfare. This simultaneously provides the opportunity to introduce a referral system between disciplines to ensure timely and adequate (oral) health support.

## Conclusions

In conclusion, this qualitative study provided detail regarding parental views on the influences on children oral health behaviours and their opinions on what further support is needed to promote children’s dental health. Their suggestions for professional oral health support can guide the development or improvement of caries preventive interventions. Important suggestions included the provision of clear oral health education using a positive approach, early referral to a dental practice, dietary regulations at school and a multidisciplinary approach in providing parental support, in which dental professionals, child health centres and other institutions work closely together to promote children’s oral health.

## Abbreviations

ACTA: Academic Centre for Dentistry Amsterdam
COREQ: consolidated criteria for reporting qualitative research
dmft: number of decayed, missing and filled primary teeth
SES: socioeconomic status
